# Aktuelles zur antirheumatischen Therapie bei Kinderwunsch, Schwangerschaft und Stillzeit

**DOI:** 10.1007/s00393-021-01095-z

**Published:** 2021-09-28

**Authors:** Celine Häfeli, Frauke Förger

**Affiliations:** Universitätsklinik für Rheumatologie und Immunologie, Inselspital Bern, Freiburgstraße 18, 3010 Bern, Schweiz

**Keywords:** Chronisch entzündliche Rheumaerkrankung, Remission, Inaktive Erkrankung, Teratogene Antirheumatika, Schwangerschaftsplanung, Chronic inflammatory rheumatic disease, Remission, Inactive disease, Teratogenic antirheumatic drugs, Pregnancy planning

## Abstract

Eine aktive chronisch entzündliche Rheumaerkrankung birgt in der Schwangerschaft Risiken für Mutter und Kind. Remission oder inaktive Erkrankung sind somit das Ziel, das im Hinblick auf die mütterliche Gesundheit und auf den Schwangerschaftsausgang zu verfolgen ist. Die antirheumatische Therapie sollte gemäß internationalen Empfehlungen bereits bei geplanter Schwangerschaft angepasst werden. Zu den erwiesenen teratogenen Antirheumatika zählen Mycophenolat, Methotrexat, Cyclophosphamid und Thalidomid, diese müssen ca. 3 Monate vor der Konzeption abgesetzt werden. Leflunomid ist ein schwaches humanes Teratogen, das vor einer Schwangerschaft abgesetzt und medikamentös ausgewaschen werden soll. Aufgrund einer unzureichenden Datenlage sollten Apremilast und JAK(Januskinase)-Inhibitoren sowie neuere Biologika in der Schwangerschaft vermieden werden. Als kompatibel mit einer Schwangerschaft gelten die Antirheumatika Hydroxychloroquin, Sulfasalazin, Azathioprin, Ciclosporin, Tacrolimus, Colchicum, nichtselektive NSAR (nichtsteroidale Antirheumatika), niedrig dosiertes Prednison/Prednisolon sowie TNF(Tumor-Nekrose-Faktor)-Hemmer. Auch in der Stillzeit sind diese Antirheumatika möglich, darüber hinaus auch andere Biologika wie Rituximab. In einem Beratungsgespräch bei Schwangerschaftsplanung sollten mit der Patientin der Nutzen sowie die internationalen Empfehlungen zur schwangerschaftskompatiblen Antirheumatikatherapie gegenüber den fetomaternalen Risiken einer aktiven Erkrankung besprochen werden, um eine gemeinsame Entscheidungsfindung zu ermöglichen.

## Abstract (en)

Active rheumatic disease is a known factor for increased fetomaternal risks during pregnancy. Remission or inactive disease should therefore be targeted to reduce these risks by using pregnancy-compatible antirheumatic drugs as recommended by international guidelines. Teratogenic antirheumatic drugs, such as mycophenolate, methotrexate, cyclophosphamide and thalidomide should be stopped about 3 months prior to conception. Leflunomide is a weak human teratogen that should be stopped and eliminated with cholestyramine prior to conception. Furthermore, drugs with limited data, such as apremilast and JAK inhibitors as well as new biologics should be avoided during gestation. Pregnancy-compatible drugs are the antirheumatic drugs hydroxychloroquine, sulfasalazine, azathioprine, cyclosporine, tacrolimus, colchicine, non-selective NSAIDs, low-dose prednisone/prednisolone and TNF inhibitors. These drugs as well as other biologics, such as rituximab can be used during lactation. In a preconception counselling visit, the benefits and the international recommendations of pregnancy-compatible antirheumatic drugs should be discussed with the patient and be weighed against the possible fetomaternal risks of an active disease to enable a shared decision-making.

Für die Betreuung von Patientinnen mit Kinderwunsch muss bedacht werden, dass eine aktive Rheumaerkrankung nicht nur Gesundheitsrisiken für die Betroffenen, sondern auch Risiken für den Ausgang der Schwangerschaft mit sich bringt. Eine antirheumatische Therapie ist deshalb auch in der Reproduktionsphase relevant. Hierzu muss die Sicherheit der Arzneimittel anhand der Datenlage vor und nach der Marktzulassung genau analysiert werden, v. a. im Hinblick auf Schädigungen des Embryos oder Feten und auf Beeinträchtigungen des Schwangerschaftsverlaufs. Eine begrenzte Anzahl von Antirheumatika gilt in der Schwangerschaft und Stillzeit als akzeptabel. Für viele neue Antirheumatika ist die Datenlage jedoch unzureichend. Internationale Empfehlungen von EULAR (European League Against Rheumatism) (2016) und ACR (American College of Rheumatology) (2020) basieren auf der jeweils vorhandenen Evidenz und helfen beim Management von Patientinnen in der Reproduktionsphase [1, 2].

## Nicht schwangerschaftskompatible „disease-modifying anti-rheumatic drugs“

Frauen mit Kinderwunsch müssen teratogene antirheumatische Medikamente rechtzeitig vor einer Konzeption stoppen. Hierzu zählen *Methotrexat (MTX), Mycophenolat-Mofetil (MMF), Cyclophosphamid* und *Thalidomid* (Tab. 1). Teratogene Medikamente verursachen bei Exposition im 1. Trimester meist ein spezifisches Fehlbildungsmuster. Insgesamt wird das teratogene Risiko eines Medikamentes immer gegenüber dem Hintergrundrisiko von etwa 3 % für das Auftreten von Fehlbildungen ohne Medikamentenexposition in der Schwangerschaft abgewogen [3]. Bei Exposition im 1. Trimester führen Thalidomid und MMF zu einem etwa 10-fach erhöhten Risiko für strukturelle Malformationen, Cyclophosphamid und MTX zu einem etwa 3‑fach erhöhten Risiko [3]. Dosis und Expositionszeitfenster spielen für die Teratogenität eine Rolle. Die oben genannten 4 teratogenen Antirheumatika müssen vor der Konzeption abgesetzt werden: MTX und Thalidomid 1 bis 3 Monate vor Konzeption, MMF > 6 Wochen vor Konzeption und Cyclophosphamid 3 Monate vor Konzeption [4]. Für die Sicherheit von Cyclophosphamid gibt es für die Behandlung von Schwangeren jenseits des 1. Trimesters Erfahrungen, die dazu beigetragen haben, dass gemäß EULAR (European League Against Rheumatism) und ACR (American College of Rheumatology) der Einsatz von Cyclophosphamid für die Behandlung von lebensbedrohlichen Erkrankungsstadien im 2. oder 3. Trimester in Betracht gezogen werden kann [1, 2].

**Table 1:** 

Substanz	Vor der Schwangerschaft	Während der Schwangerschaft
*Konventionelle Antirheumatika*		
Hydroxychloroquin	++	++
Sulfasalazin	++ (plus Folsäure)	++ (plus Folsäure)
Azathioprin	++	++
Ciclosporin, Tacrolimus	+ (RR-Kontrolle)	+ (RR-Kontrolle)
Colchicum	++	++
Prednison/Prednisolon	+	+ (Ziel ≤ 10 mg/Tag)
Nichtselektive NSAR	+ (Stopp bei Subfertilität)	+ (Stopp im 3. Trimester)
*Schwangerschaftskompatible Biologika*		
Certolizumab	++	++
Infliximab, Etanercept, Adalimumab, Golimumab	+	+ (im 3. Trimester mehrere Halbwertszeiten vor Entbindungstermin stoppen)
Rituximab	+ (bis zur Konzeption)	+ (fortführen bei lebens-/organbedrohender Erkrankung)
*Biologika mit limitierter Sicherheitsdatenlage in der Schwangerschaft*		
Anakinra, Canakinumab, Belimumab, Abatacept, Tocilizumab, Secukinumab, Ustekinumab	+ (bis zur Konzeption)	Nein, bei fehlender therapeutischer Alternative ist ein individualisiertes Vorgehen zu erwägen
*Nicht schwangerschaftskompatible Antirheumatika*		
Methotrexat	Stopp 1 bis 3 Monate vor Konzeption, Folsäure geben	Nein
Mycophenolat-Mofetil, Mycophenolsäure	Stopp > 6 Wochen vor Konzeption	Nein
Cyclophosphamid	Stopp 3 Monate vor Konzeption	Nein im 1. Trimester + im 2. und 3. Trimester bei lebens-/organbedrohender Erkrankung
Thalidomid	Stopp 1 bis 3 Monate vor Konzeption	Nein
Leflunomid	Stopp vor Konzeption, Washout bei nachweisbarem Spiegel	Nein
Tofacitinib, Baricitinib	Stopp vor Konzeption (s. Herstellerinfo)	Nein

*RR* Blutdruck, *NSAR* nichtsteroidale Antirheumatika

Als nicht schwangerschaftskompatibles DMARD („disease-modifying anti-rheumatic drug“) gilt auch *Leflunomid*, das sich in präklinischen Tierstudien bei Ratten und Kaninchen in humantherapeutischen Dosierungen als teratogen erwies. Jedoch gibt es unter den zugelassenen Leflunomid-Dosen beim Menschen nach Auswaschen kein erhöhtes Risiko für grobstrukturelle Fehlbildungen bei Exposition im 1. Trimester [1]. Die Evidenz beruht auf der prospektiven OTIS-Kohortenstudie von 64 Leflunomid-behandelten Patientinnen, von denen 95,3 % ein Cholestyramin-Washout erhielten. Die Malformationsrate der Neugeborenen war in dieser Studie vergleichbar hoch wie die der nicht exponierten Kontrollgruppen [5]. Auch in 3 weiteren prospektiven Datenbankkohorten mit 51 (Kanada), 47 (Embryotox-Deutschland) und 206 (Sanofi Pharmakovigilanz) Fällen von Leflunomid-Expositionen im 1. Trimester – allerdings ohne Kontrollgruppe – zeigten sich keine erhöhten Malformationsraten [6–8]. Der Anteil der Patientinnen mit Cholestyramin-Washout war hier wesentlich geringer und lag bei 34–45 %. Die Autoren der Embryotox-Studie machen darauf aufmerksam, dass die vom Hersteller herausgegebenen Empfehlungen zu vorsichtig wären, nämlich die einer Leflunomid-Konzentration von unter 0,02 mg/l (diese liegt 100-fach unter dem „no-effect level“ in präklinischen Studien) bzw. ein Absetzen 2 Jahre vor geplanter Konzeption [7]. Weitergefasste Nachweisgrenzen (z. B < 2 mg/l) könnten somit akzeptabel sein. Die internationalen Empfehlungen bezüglich Leflunomid und Schwangerschaft sind also insgesamt vorsichtig. Es wird ein Absetzen von Leflunomid vor einer Schwangerschaft empfohlen und bei noch nachweisbarem Spiegel ein Auswaschverfahren mit Cholestyramin [1, 2].

Für *JAK(Januskinase)-Inhibitoren* ist die Datenlage zur Sicherheit in der Schwangerschaft unzureichend. In präklinischen Untersuchungen erwiesen sich Tofacitinib und Baricitinib bei Ratten und Kaninchen in deutlich höheren als den humantherapeutischen Dosierungen als teratogen und hatten bei weiblichen Tieren Auswirkungen auf die Fertilität. Dagegen zeigten die Pharmakovigilanzdaten von Pfizer aus Interventionsstudien, dass bei 74 Schwangerschaften mit mütterlicher Tofacitinib-Exposition die Abort- und Malformationsrate nicht höher war als die der Referenzpopulation [9]. Schwächen solcher Pharmakovigilanzdaten sind unter anderem fehlende Daten von Schwangerschaftsausgängen (hier 32 von 74) und fehlende Kontrollgruppen. Sowohl EULAR als auch ACR empfehlen ein Absetzen von JAK-Inhibitoren vor geplanter Konzeption [2]. Die Hersteller empfehlen bei geplanter Konzeption eine 1‑wöchige (Baricitinib) bis 4‑wöchige (Tofacitinib, Upadacitinib) Pause unter effektiver Empfängnisverhütung nach letzter Dosis (Tab. 1).

Absetzen von teratogenen Antirheumatika vor einer Schwangerschaft wird empfohlen

Für *Apremilast* zeigen präklinische Studien in Mäusen und Affen, dass es zu einer dosisabhängigen embryofetalen Entwicklungstoxizität kommt [1]. Bei bis zum 3,5-Fachen der klinischen Expositionsdosis zeigten sich bei Affen keine negativen Auswirkungen auf die fetale Entwicklung oder Fehlbildungen [1]. Die Datenlage bei menschlichen Schwangerschaften ist unzureichend, deshalb wird ein Absetzen von Apremilast vor einer geplanten Konzeption empfohlen (Tab. 1) [1].

## Schwangerschaftsverträgliche konventionelle Antirheumatika

Kortikosteroide wie *Prednison* oder *Prednisolon* können zur Kontrolle der Krankheitsaktivität in der Schwangerschaft eingesetzt werden (Tab. 1). Im Falle einer Langzeittherapie gilt es jedoch, die niedrigste effektive Dosis zu wählen, um Risiken einer möglichen Infektion und/oder Frühgeburt zu reduzieren [10, 11]. Denn bei Prednison-Äquivalenzdosen über 10 mg/Tag erhöht sich das Frühgeburtsrisiko [11, 12]. Kurzzeitige intravenöse oder orale Glukokortikoidboli können in der Schwangerschaft appliziert werden, sollten jedoch rasch unter 20 mg pro Tag reduziert werden [1, 2]. Um eine möglichst niedrige Glukokortikoiddosis in der Schwangerschaft einsetzen zu können, wird die Kombination mit schwangerschaftsverträglichen Basismedikamenten oder Biologika empfohlen [4].

Fluorierte Glukokortikoide wie *Dexamethason* oder *Betamethason* werden im Vergleich zu Prednisolon deutlich geringer in der Plazenta metabolisiert und daher für fetale Indikationen in der Schwangerschaft – wie beispielsweise zur Lungenreifung oder zur Therapie eines kongenitalen Herzblocks – gegeben.

Nichtselektive *NSAR* (nichtsteroidale Antirheumatika) wie Ibuprofen und Diclofenac können im 1. und 2. Trimester eingesetzt werden, von COX-2-Hemmern wird in der Schwangerschaft hingegen abgeraten (Tab. 1) [1, 2]. Da NSAR Ovulationsstörungen verursachen können und so zu einer möglichen Subfertilität führen – dies wurde v. a. für COX-2-Hemmer nachgewiesen [13] – sollten NSAR bei Fertilitätsproblemen eher vermieden werden. Selten kann es bei Einsatz von NSAR nach der 20. Schwangerschaftswoche zu fetalen Nierenfunktionsstörungen mit Oligohydramnion kommen, die FDA (US Food & Drug Administration) hat 2020 diesbezüglich eine Warnung herausgegeben. Im 3. Trimester dürfen NSAR nicht mehr gegeben werden, da sie zu einem vorzeitigen Verschluss des Ductus arteriosus führen können.

NSAR sollten bei Fertilitätsproblemen eher vermieden werden

Die Basismedikamente *Hydroxychloroquin, Chloroquin, Sulfasalazin, Azathioprin, Ciclosporin, Tacrolimus* und *Colchicum* sind schwangerschaftskompatibel und sollten zum Erhalt einer stabilen inaktiven Erkrankung in der Schwangerschaft fortgeführt werden (Tab. 1) [1, 2]. Idealerweise werden diese DMARDs schon in der Planungsphase einer Schwangerschaft begonnen. Beachtet werden sollte die Empfehlung einer zusätzlichen Folsäuregabe bei Sulfasalazin und ein Blutdruckmonitoring bei den Calcineurininhibitoren Ciclosporin und Tacrolimus.

Hervorzuheben ist die multizentrische prospektive PATCH(Preventive Approach to Congenital Heart Block with Hydroxychloroquine)-Studie, die den Effekt von *Hydroxychloroquin* (HCQ) auf das Wiederholungsrisiko eines intrauterinen kongenitalen Herzblocks (CHB) bei Feten von schwangeren Frauen mit Anti-Ro-Antikörper ± Anti-La-Antikörper untersucht [14]. Hintergrund dieser Studie ist das Risiko eines CHB von etwa 2 % bei Anti-SSA- und Anti-SSB-Antikörper-positiven Schwangeren, wenn die Mutter erstmalig schwanger ist oder in vorausgegangenen Schwangerschaften kein Kind mit CHB hatte [14]. Bei Anti-SSA/Anti-SSB-positiven Müttern von Kindern mit CHB ist in einer nächsten Schwangerschaft das Wiederholungsrisiko für einen CHB höher und liegt bei etwa 18 % [14]. Die PATCH-Studie zeigte, dass HCQ (400 mg pro Tag), das bereits vor der 10. Schwangerschaftswoche gegeben und während der gesamten Schwangerschaft fortgeführt wurde, das Wiederholungsrisiko eines CHB um über 50 % (7,4 % anstatt 18 %) reduziert [14].

Weiterhin belegt eine Fall-Kontroll-Studie, dass HCQ bei Schwangeren mit Positivität für Anti-Ro-Antikörper ± Anti-La-Antikörper auch das Risiko eines kutanen neonatalen Lupus signifikant verringert [15].

In Anbetracht der Hydroxychloroquin-Studien bei COVID-19 rückte auch die mögliche Kardiotoxizität mit Entwicklung eines QT-Syndroms in den Fokus. In einer Untergruppenanalyse der PATCH-Studie wurden die EKGs (Elektrokardiogramm) der Hydroxychloroquin-exponierten Neugeborenen hinsichtlich des QT-Intervalls untersucht, und dabei wurde festgestellt, dass es keine Korrelation zwischen dem Hydroxychloroquin-Spiegel im Nabelschnurblut und dem QT-Intervall gab [16]. Die Inzidenz einer QT-Intervall-Verlängerung war mit 11 % niedrig und ohne Symptome [16].

Eine systematische Übersichtsarbeit zeigte, dass das Risiko für Augenauffälligkeiten unter den 331 Kindern mit In-utero-Hydroxychloroquin/Chloroquin-Exposition und nachfolgenden augenärztlichen Untersuchungen gering bis nicht existent ist [17]. Jedoch sind bei diesen Studien in der Regel nicht alle modernen Screeningtests zum Einsatz gekommen [17, 18].

## Generelle Aspekte zu Biologika in der Schwangerschaft

Während der Schwangerschaft passieren mütterliche Immunglobuline (Ig) vom IgG-Isotyp die Plazentaschranke. Dieser Transport beginnt im 2. Trimester und nimmt zum Ende des 3. Trimesters kontinuierlich zu. So beträgt der Anteil von mütterlichem IgG im fetalen Blut zwischen der 17. und 22. Schwangerschaftswoche etwa 5–10 %, um die 32. Schwangerschaftswoche etwa 50 %, und am Geburtstermin liegt er über 100 % [19, 20]. Somit sind beim reifen Neugeborenen höhere IgG-Spiegel messbar als bei der Mutter. Dieser aktive Transport erfolgt über Bindung des konstanten Fc-Anteils des IgG-Moleküls an neonatalen Fc-Rezeptoren (FcRn) auf Synzytiotrophoblastenzellen der Plazenta [21]. Am stärksten ist dieser aktive maternofetale transplazentare IgG-Transport für die IgG1-Subklasse, gefolgt von IgG4, IgG3 und IgG2 [20]. Bei den Biologika handelt es sich in der Regel um komplette monoklonale IgG1-Antikörper. Werden diese in der Schwangerschaft appliziert, so unterliegen sie demselben transplazentaren FcRn-vermittelten Transport wie andere IgG1-Moleküle. Entsprechend sind bei intrauteriner Infliximab- oder Adalimumab-Exposition im 3. Trimester die Konzentrationen dieser TNF(Tumor-Nekrose-Faktor)-Inhibitoren im Serum der Neugeborenen etwa 50–60 % höher als die der Mütter [22]. Rezeptorfusionsproteine wie Etanercept zeigen dagegen einen geringeren transplazentaren FcRn-vermittelten Transport; noch geringer bis nicht existent ist der transplazentare Transfer von Certolizumab, das aufgrund des fehlenden Fc-Anteils nicht an den FcRn bindet [23–25].

## Tumor-Nekrose-Faktor-Inhibitoren in der Schwangerschaft

TNF-Inhibitoren zeigen kein erhöhtes Malformationsrisiko – weder in präklinischen Untersuchungen noch bei mütterlicher Exposition während der Schwangerschaft. Dies belegen Daten von rund 2500 Schwangerschaften aus Kohortenstudien, Registerdaten und zahlreiche größere Fallserien, die in die EULAR-Empfehlungen eingeflossen sind [2]. In den letzten Jahren kamen noch einige Publikationen hinzu, von denen hier die wichtigsten kurz erwähnt werden. Insgesamt gibt es die meisten Daten zu folgenden TNF-Hemmern: Adalimumab, Infliximab, Etanercept und Certolizumab [26].

TNF-Inhibitoren zeigen kein erhöhtes Malformationsrisiko

Eine kanadische populationsbasierte Kohortenstudie zeigte, dass TNF-Inhibitoren in den 120 untersuchten Schwangerschaften nicht mit einem Frühgeburtsrisiko oder einem erniedrigten Geburtsgewicht von Neugeborenen assoziiert sind [27].

Eine Metaanalyse (9 Studien) verglich Patientinnen mit vs. ohne TNF-Inhibitor-Exposition in der Schwangerschaft und zeigte, dass TNF-Inhibitoren keine erhöhten Risiken für Schwangerschaftskomplikationen inklusive kongenitale Anomalien, Aborte oder Frühgeburten mit sich bringen [28].

Pharmakovigilanzdaten des *Certolizumab*-Herstellers zeigten, dass unter den 1392 prospektiv berichteten Schwangerschaften unter Certolizumab-Exposition – in etwa 40 % lag eine Exposition in allen 3 Trimestern vor – keine erhöhte Malformationsrate, keine erhöhte Rate für Schwangerschaftskomplikationen wie Fehlgeburten, Frühgeburten oder erniedrigtes Geburtsgewicht auftrat [29].

Ein höheres Evidenzlevel für die Sicherheit von TNF-Inhibitoren in der Schwangerschaft wird in der prospektiven Kohortenstudie der OTIS-Gruppe deutlich, bei der 257 schwangere Patientinnen mit rheumatoider Arthritis (RA) und Morbus Crohn unter *Adalimumab* vs. 120 Patientinnen mit RA und Morbus Crohn ohne Adalimumab vs. 225 Schwangere ohne Erkrankung und Therapie verglichen wurden [30]. Es zeigte sich unter Adalimumab keine erhöhte Rate von Frühgeburten und keine erhöhte Rate von Malformationen verglichen mit der nicht exponierten Kontrollgruppe [30].

Während die EULAR-Empfehlungen aufgrund der fehlenden Daten zu *Golimumab* noch vorsichtig waren und zu einem alternativen TNF-Inhibitor in der Schwangerschaft mit besserer Datenlage rieten [2], setzen die ACR-Empfehlungen von 2020 nun Golimumab auf die gleiche Stufe wie Infliximab, Etanercept und Adalimumab [1]. Pharmakovigilanzdaten des Herstellers zu 208 Schwangerschaften mit Golimumab-Exposition wurden auf einem vergangenen ACR-Kongress präsentiert, dabei zeigte sich keine erhöhte Rate für kongenitale Malformation oder Aborte verglichen mit der Allgemeinbevölkerung [31].

All dies sind die Grundlagen dafür, dass sowohl die EULAR- als auch die ACR-Richtlinien die Fortführung der TNF-Inhibitoren *Infliximab, Adalimumab, Golimumab* und *Etanercept* im 1. und 2. Trimester empfehlen, um damit eine inaktive Erkrankung zu erhalten (Tab. 1) [1, 2]. Wenn möglich – d. h. bei inaktiver stabiler Erkrankung – sollten diese TNF-Inhibitoren im 3. Trimester pausiert werden. Bei Patientinnen mit rheumatoider Arthritis ist bei niedriger Aktivität bzw. inaktiver Erkrankung ein Pausieren der TNF-Hemmer nach 20. Schwangerschaftswoche möglich und nicht mit einem nachfolgenden Schub assoziiert [32, 33]. Im Falle von verbleibender Krankheitsaktivität sollte jedoch eine Fortführung dieser TNF-Inhibitoren im 3. Trimester erwogen werden [1, 2]. *Certolizumab* kann während der gesamten Schwangerschaft eingesetzt werden [1, 2].

## Rituximab und Belimumab in der Schwangerschaft

In präklinischen Untersuchungen zur Reproduktionstoxizität in Affen zeigten sich bei *Rituximab*-Exposition in der Schwangerschaft keine Fehlbildungen bei den Nachkommen. Allerdings ist Rituximab ein kompletter IgG-Antikörper und wird in der Schwangerschaft transplazentar zum Föten transportiert. So kann es bei mütterlicher Rituximab-Therapie im 2. und 3. Trimester zu einer B‑Zell-Depletion und Lymphopenie bei Neugeborenen kommen [2]. Rituximab kann gemäß internationalen Richtlinien bis zur Konzeption gegeben werden und bei dringenden Indikationen auch während der Schwangerschaft (Tab. 1) [1, 2].

*Belimumab* ist ein rekombinantes IgG-Molekül gegen das lösliche B‑Lymphozyten-Stimulator-Protein (BLyS). Präklinische Daten zeigen keine Embryo- oder Fetotoxizität. Aufgrund seiner Struktur ist Belimumab plazentagängig. In einem laufenden prospektiven Register können Schwangerschaften unter Belimumab eingeschlossen werden (bei Belimumab-Exposition innerhalb 4 Monate vor Konzeption oder während der Schwangerschaft, E‑Mail: belimumab.pregnancy@ppdi.com). Die begrenzte Datenlage suggeriert bisher kein erhöhtes Risiko für Malformationen, jedoch bleibt die Auswertung des Registers abzuwarten [2]. Gemäß ACR 2020-Empfehlungen kann Belimumab bis zur Konzeption fortgeführt werden (Tab. 1) [4]. Sollte es keine Therapiealternativen mit besserer Evidenzlage in der Schwangerschaft geben, kann der Nutzen einer fortgeführten Belimumab-Therapie in der Schwangerschaft mit den Risiken einer unkontrollierten SLE(systemischer Lupus erythematodes)-Erkrankung abgewogen werden [4].

## Biologika mit unzureichender Datenlage in der Schwangerschaft

Informationen zu präklinischen Daten von den Herstellern zeigen, dass *Ustekinumab, Secukinumab, Abatacept, Tocilizumab, Sarilumab, Anakinra *und* Canakinumab* keine Embryo- oder Fötotoxizität und keine unerwünschten Wirkungen bezüglich des fetalen oder neonatalen Wachstums aufweisen. Insbesondere zeigen diese präklinischen Untersuchungen keine substanzbezogene Teratogenität auf, was wahrscheinlich an dem fehlenden maternoembryonalen Transfer dieser IgG-basierten Biologika während des 1. Trimesters liegt. Wie oben erwähnt, gibt es erst ab dem 2. Trimester einen maternofetalen IgG-Transport via neonatalen Fc-Rezeptoren, der im Falle einer Therapie während dieser Trimester eine Einwirkung dieser Biologika auf den Feten und das Neugeborene überhaupt erst ermöglicht [21].

Die Datenlage zu diesen Biologika wurde ausführlich in den EULAR 2016-Empfehlungen und den ACR 2020-Empfehlungen gesichtet und dargestellt [2, 4]. Bei einigen dieser Biologika erschienen in den letzten Jahren größere Fallzahlen aus den Sicherheitsdatenbanken der Herstellerfirmen:

Größere Zahlen wurden im letzten Jahr zu *Ustekinumab* und Schwangerschaft veröffentlicht. Der Hersteller publizierte Daten aus der Sicherheitsdatenbank in Abstract-Form: 478 Schwangerschaften bei mütterlicher Ustekinumab-Exposition bis 3 Monate vor Konzeption oder während der Schwangerschaft [34]. Bei diesen Schwangerschaften kam es in 71,3 % zu Lebendgeburten, bei 18,4 % zu Aborten, bei 10,3 % war der Ausgang unbekannt. Malformationen traten in 3,8 % der Ustekinumab-exponierten Schwangerschaften auf und damit nicht häufiger als in der Allgemeinbevölkerung. Ebenso zeigen die PSOLAR-Registerdaten, dass bei 70 Patientinnen mit Ustekinumab-Therapie während der Schwangerschaft die Rate für Malformationen (1,8 %) und Spontanaborte (14,3 %) nicht erhöht ist [35].

Zu *Secukinumab* wurde aus der globalen Novartis-Sicherheitsdatenbank der Ausgang von 238 Schwangerschaften mit mütterlicher Exposition, davon 65 % mit Therapie im 1. Trimester, berichtet [36]. Leider war der Ausgang der Schwangerschaft bei 119 unbekannt, unter den Schwangerschaften mit bekanntem Ausgang waren die Abort- und Malformationsrate nicht erhöht [36].

Zu *Tocilizumab* lieferte die globale Sicherheitsdatenbank von Roche die meisten Daten [37]. Die Analyse zeigte, dass bei 180 prospektiven Schwangerschaften sowie bei 108 retrospektiven Schwangerschaften, in denen die Patientinnen präkonzeptionell oder während der Schwangerschaft Tocilizumab nahmen, keine erhöhte Rate von kongenitalen Malformationen auftrat – insbesondere keine spezifischen Malformationsmuster [37]. Aktuelle Daten zeigten zudem die Anwendung von Tocilizumab im 2. und 3. Trimester bei schwangeren Frauen, die an COVID-19 erkrankten – bei 12 berichteten Fälle wurden keine negativen Auswirkungen von Tocilizumab auf den Fetus bzw. das Neugeborene beschrieben [38].

Insgesamt gibt es für *Ustekinumab, Secukinumab, Abatacept, Tocilizumab, Sarilumab, Anakinra* sowie *Canakinumab* keine ausreichende Datenlage. Deshalb lautet die Empfehlung hier, diese Biologika bei positivem Schwangerschaftstest abzusetzen (Tab. 1) [1, 2]. Falls es jedoch zur Kontrolle der Krankheitsaktivität keine schwangerschaftskompatible Alternative gibt, ist der klinische Nutzen dieser Biologika gegenüber der limitierten Datenlage in der Schwangerschaft abzuwägen [1]. Wichtig in diesem Zusammenhang ist, dass eine erhöhte Krankheitsaktivität in der Regel ein Risikofaktor für eine Frühgeburt und für ein Neugeborenes mit erniedrigtem Geburtsgewicht ist [39–41].

## Biologika in der Stillzeit

In den ersten 6 Monaten nach der Geburt besteht oft ein erhöhtes Risiko für Krankheitsschübe. Die meisten Patientinnen stillen in dieser Phase, und es stellt sich die Frage nach einer stillkompatiblen antirheumatischen Therapie. Auch diesbezüglich gibt es in den Empfehlungen der ACR von 2020 Änderungen [4]. Neu ist, dass alle TNF-Hemmer sowie Rituximab als stillkompatibel gelten (ACR 2020: „strongly recommended“) [4].

Alle TNF-Hemmer sowie Rituximab gelten als stillkompatibel

Auch andere Biologika wie Anakinra, Belimumab, Abatacept, Tocilizumab, Secukinumab sowie Ustekinumab können in der Stillzeit erwogen werden (Tab. 1) (ACR 2020: „conditionally recommended“) [4]. Dabei ist die Datenlage zu jedem einzelnen Biologikum limitiert oder nicht existent [42, 43]. Jedoch leiten sich diese Empfehlungen von der Pharmakokinetik der monoklonalen Antikörper ab. So wird in der Regel v. a. IgA und nicht IgG in die Muttermilch sezerniert [44]. Zudem besteht im Falle einer oralen Aufnahme von Biologika aufgrund der gastrointestinalen Proteolyse keine relevante Bioverfügbarkeit für diese Substanzen [45]. Die kalkulierten relativen kindlichen Dosen liegen somit unter den als nicht bedenklich erachteten 10 %, in der Regel sogar deutlich tiefer [43, 46, 47].

## Kindliche Gesundheit nach In-utero-Biologika-Exposition

### Infektionen

Prospektive Verlaufsuntersuchungen im 1. Lebensjahr von 229 Adalimumab-exponierten Kindern zeigten keine erhöhte Rate von schweren oder opportunistischen Infektionen gegenüber einer nicht exponierten Kontrollgruppe [30].

Insgesamt zeigen diese Daten mehrheitlich keine erhöhte Infektrate bei Kindern nach In-utero-Biologikaexposition

Eine weitere große prospektive Beobachtungsstudie bei 1490 Schwangeren mit entzündlichen Darmerkrankungen untersuchte das Outcome von 1431 Kindern, davon 616 nach intrauteriner Biologikaexposition (99 Infliximab, 66 Adalimumab, 33 Certolizumab, 4 Golimumab, 22 Vedolizumab, 4 Natalizumab, 7 Ustekinumab) [48]. Die Nachfolgeuntersuchungen der Kinder in den ersten 12 Monaten zeigten vergleichbare Entwicklungsmeilensteine wie nicht-Biologika-exponierte Kinder, ebenso fand sich keine erhöhte Rate von schweren oder nichtschweren Infektionen bei Biologika-exponierten Kindern (sowohl in der Gesamtgruppe als auch in der TNF-Hemmer-Untergruppe) [48]. Auch eine retrospektive Untersuchung von 388 Kindern mit In-utero-TNF-Hemmer-Exposition wies bei einer Langzeitbeobachtung von etwa 2 Jahren keine erhöhte Rate an schweren Infektionen gegenüber einer nicht exponierten Vergleichsgruppe auf [49].

### Impfungen

Bei Einsatz von Biologika in der Schwangerschaft soll gemäß EULAR- und BSR(British Society of Rheumatology)-Empfehlungen bei den exponierten Kindern in den ersten 6 bis 7 Monaten keine Lebendimpfung durchgeführt werden [2, 50]. Hintergrund dieser Empfehlung ist der oben beschriebene transplazentare Transport der monoklonalen IgG-Antikörper, der bei Einsatz dieser Biologika im 3. Trimester zu therapeutischen Medikamentenspiegeln beim Neugeborenen führen kann [2]. Folgt man jedoch den Empfehlungen von EULAR mit Absetzen von Infliximab und Adalimumab in der 20. Schwangerschaftswoche, so sind die im Nabelschnurblut der Neugeborenen gemessenen Medikamentenspiegel gering bis nicht messbar [51].

Alarmierend war ein Fallbericht einer tödlich endenden BCG(Bacillus Calmette-Guérin)-Lebendimpfung eines 3 Monate alten Kindes einer Mutter mit entzündlicher Darmerkrankung und Infliximab-Behandlung während der gesamten Schwangerschaft im Jahr 2010 [52]. Dennoch zeigte eine retrospektive Fallanalyse von 15 Kindern, die im 2. und 3. Trimester der Schwangerschaft einer TNF-Hemmer-Behandlung ausgesetzt waren, dass nach einer BCG-Impfung keine schweren Komplikationen auftraten [53]. Weiterhin fand eine Studie zum Impfansprechen bei 40 Kindern von Müttern mit entzündlichen Darmerkrankungen und Biologikatherapie in der Schwangerschaft, dass eine in den ersten 6 Monaten gegebene orale Rotavirus-Lebendimpfung mehrheitlich gut verlief und nur bei 17,5 % der Impflinge leichte Impfreaktionen (Fieber, Diarrhö) vorkamen – eine ähnliche Impfreaktionsrate wie bei historischen Kontrollen [54]. Weiterhin zeigte diese Studie, dass Biologika-exponierte Kinder nach einer Impfung gegen Haemophilus influenzae B und Tetanustoxin (*n* = 38) eine vergleichbar gute Impfantwort entwickelten wie nicht exponierte Kinder [54].

Zusammenfassend kann man sagen, dass gemäß internationalen Guidelines bei Biologika-exponierten Kindern (insbesondere bei intrauteriner Exposition mit kompletten monoklonalen Antikörpern) keine Lebendimpfung in den ersten 6 bis 7 Lebensmonaten gegeben werden sollte [2, 50]. Dies gilt v. a. für die in Deutschland und Österreich in diesem Zeitraum vorgesehene Rotavirus-Impfung. Die BCG-Impfung steht weder in Deutschland noch in Österreich oder der Schweiz auf dem Plan für Standardimpfungen. Unserer persönlichen Einschätzung nach führt der Einsatz des Fc-freien TNF-Hemmers Certolizumab in der Schwangerschaft aufgrund der minimalen bis nicht vorhandenen Plazentagängigkeit nicht zu Einschränkungen im Hinblick auf Lebendimpfungen der intrauterin exponierten Kinder.

## Fazit für die Praxis

- Insgesamt nimmt die Evidenzlage für die Sicherheit einer effizienten antirheumatischen Therapie in der Schwangerschaft zu. Dies verbessert sowohl das mütterliche wie auch das kindliche Outcome.
- Bei Patientinnen mit rheumatischen Erkrankungen sollte es sich immer um eine geplante Schwangerschaft in einer inaktiven Krankheitsphase handeln.
- Die antirheumatische Therapie muss rechtzeitig vor einer Schwangerschaft oder Konzeption angepasst werden.
- In einem Aufklärungsgespräch vor geplanter Schwangerschaft gilt es, mit der Patientin den Nutzen einer antirheumatischen Therapie für den Erhalt einer niedrigen Krankheitsaktivität gegenüber den potenziellen Risiken wie Frühgeburt bei aktiver Erkrankung oder schlechter Datenlage eines Medikamentes abzuwägen.
